# Epigenetic and transcriptional analysis reveals a core transcriptional program conserved in clonal prostate cancer metastases

**DOI:** 10.1002/1878-0261.12923

**Published:** 2021-03-11

**Authors:** Tesa M. Severson, Yanyun Zhu, Angelo M. De Marzo, Tracy Jones, Jonathan W. Simons, William G. Nelson, Srinivasan Yegnasubramanian, Matthew L. Freedman, Lodewyk Wessels, Andries M. Bergman, Michael C. Haffner, Wilbert Zwart

**Affiliations:** ^1^ Division of Oncogenomics Oncode Institute Netherlands Cancer Institute Amsterdam the Netherlands; ^2^ Division of Molecular Oncogenesis Oncode Institute Netherlands Cancer Institute Amsterdam the Netherlands; ^3^ Sidney Kimmel Comprehensive Cancer Center Department of Pathology Brady Urological Institute Johns Hopkins School of Medicine Baltimore MD USA; ^4^ Department of Pathology Johns Hopkins School of Medicine Baltimore MD USA; ^5^ Prostate Cancer Foundation Santa Monica CA USA; ^6^ Sidney Kimmel Comprehensive Cancer Center Johns Hopkins School of Medicine Baltimore MD USA; ^7^ Department of Medical Oncology Dana‐Farber Cancer Institute Harvard Medical School Boston MA USA; ^8^ The Eli and Edythe L. Broad Institute Cambridge MA USA; ^9^ Department of Medical Oncology Netherlands Cancer Institute Amsterdam the Netherlands; ^10^ Divisions of Human Biology and Clinical Research Fred Hutchinson Cancer Research Center Seattle WA USA; ^11^ Department of Pathology University of Washington Seattle WA USA; ^12^ Department of Pathology Johns Hopkins School of Medicine Baltimore MD USA; ^13^ Laboratory of Chemical Biology and Institute for Complex Molecular Systems Department of Biomedical Engineering Eindhoven University of Technology Eindhoven the Netherlands

**Keywords:** ChIP‐seq, cistrome, epigenomics, metastasis, prostate cancer, transcriptomics

## Abstract

The epigenomic regulation of transcriptional programs in metastatic prostate cancer is poorly understood. We studied the epigenomic landscape of prostate cancer drivers using transcriptional profiling and ChIP‐seq in four clonal metastatic tumors derived from a single prostate cancer patient. Our epigenomic analyses focused on androgen receptor (AR), which is a key oncogenic driver in prostate cancer, the AR pioneer factor FOXA1, chromatin insulator CCCTC‐Binding Factor, as well as for modified histones H3K27ac and H3K27me3. The vast majority of AR binding sites were shared among healthy prostate, primary prostate cancer, and metastatic tumor samples, signifying core AR‐driven transcriptional regulation within the prostate cell lineage. Genes associated with core AR‐binding events were significantly enriched for essential genes in prostate cancer cell proliferation. Remarkably, the metastasis‐specific active AR binding sites showed no differential transcriptional output, indicating a robust transcriptional program across metastatic samples. Combined, our data reveal a core transcriptional program in clonal metastatic prostate cancer, despite epigenomic differences in the AR cistrome.

## Graphical abstract

Epigenomic and transcriptomic profiling of 4 clonal metastatic samples taken from a patient with prostate cancer during a single autopsy reveals sample‐shared/specific Androgen Receptor (AR) binding sites with enhancer activity. Sample‐shared AR sites are conserved across disease transition states, which are enriched in genes essential for prostate cancer proliferation. Remarkably, genes associated with active metastatic sample‐specific AR binding sites showed no differential transcriptional output.

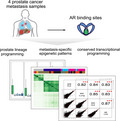

**Abbreviations**
AR: Androgen Receptor
ATRX: ATRX Chromatin Remodeler
ChIP‐seq: Chromatin Immunoprecipitation Sequencing
CTCF: CCCTC‐Binding Factor
ESR1: Estrogen Receptor 1
ETS: ETS Transcription Factor ELK1
EZH2: Enhancer Of Zeste 2 Polycomb Repressive Complex 2 Subunit
FFPE: Formalin‐Fixed Paraffin‐Embedded
FOS: Fos Proto‐Oncogene, AP‐1 Transcription Factor Subunit
FOXA1: Forkhead Box A1
FOXA2: Forkhead Box A2
H3K27ac: Histone 3 lysine acetylation
H3K27me3: Histone 3 lysine trimethylation
HDAC: Histone Deacetylase
JUN: Jun Proto‐Oncogene, AP‐1 Transcription Factor Subunit
mCRPC: metastatic castration‐resistant prostate cancer
NE: Neuroendocrine
NR3C1 (GR): Nuclear Receptor Subfamily 3 Group C Member 1
PDX: Patient‐Derived Xenograft
PGR: Progesterone Receptor
PSA: Prostate Serum Antigen
PTEN: Phosphatase And Tensin Homolog
SPOP: Speckle Type BTB/POZ Protein
TP53: Tumor Protein P53

## 1 Introduction

In metastatic castration‐resistant prostate cancer (mCRPC), under the selective pressure of low circulating testosterone levels, tumors typically respond by restoring the Androgen Receptor (AR) pathway by means of activating mutations or splice variants of the AR [1, 2, 3, 4], amplification of the gene itself [5, 6], or its associated enhancer [7]. In addition, changes in the genome‐wide chromatin interactome of the AR (hereafter referred to its ‘cistrome’) have been characterized in cell line models, primary tumors, and mCRPC [8, 9, 10, 11]. Interestingly, in primary prostate cancers of different patients, AR chromatin interactions have been shown to be highly variable [9], suggesting a high level of cistrome heterogeneity in primary disease. Although the genomic landscape of mCRPC is heterogenous, numerous studies have demonstrated that genomic driver alterations are shared between different metastatic sites in a given patient [12, 13, 14, 15, 16]. These findings establish that distant metastases likely arise from a single cell clone in the primary tumor and show a high level of genetic similarity, irrespective of their anatomic location [15, 17]. Despite these well‐established genetic similarities, little is known about the AR cistrome and its surrounding epigenome, along with the corresponding transcriptome between different metastatic sites. Prostate cancer is considered an epigenetic disease [18] and multiple epigenetic drugs (e.g., HDAC and Enhancer Of Zeste 2 Polycomb Repressive Complex 2 Subunit inhibitors) are in clinical development for the treatment of mCRPC [19], ClinicalTrials.gov NCT03480646. For genomic analyses in mCRPC, a biopsy procedure is performed on an accessible metastatic lesion [20, 21] and the question remains whether sampling bias would impact the clinical decision‐making process.

**Fig. 1:**
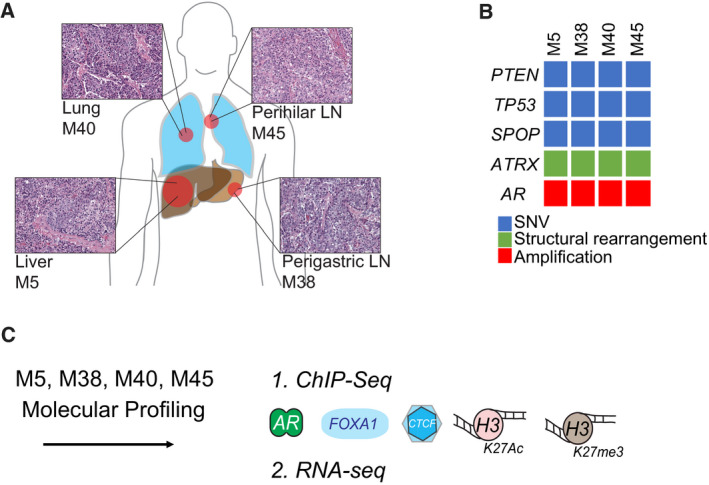
Anatomically distinct metastases share histomorphological features and driver alterations. (A) Schematic of locations of the metastasis samples examined along with their H&E stainings indicating cell nuclei in purple. (B) Schematic indicating SNV, structural rearrangement, and amplification alteration for the driver genes identified to be shared among the different metastases samples. (C) Molecular data collected for metastases samples, 1.) ChIP‐seq for AR, FOXA1, CTCF, H3K27ac, H3K27me3, and 2.) RNA‐seq.

Previously, Haffner *et al*. [15] described the clonal genomic relationship between distant metastases and the primary tumor from a single patient (Fig. 1A). Importantly, this study revealed that the vast majority of genomic alterations are present in all distant metastases, thereby demonstrating limited interlesional heterogeneity on the genomic level. In the backdrop of these genomically highly similar metastases, we aimed to investigate in specimens from the same patient, if epigenomic and downstream transcriptional programming differ between anatomically distinct metastatic sites.

In order to comprehensively survey the epigenome, we generated ChIP‐seq data for AR, its pioneer factor FOXA1 [22], the chromatin insulator CCCTC‐Binding Factor (CTCF) [23], histone modification H3K27ac demarcating active promoters and enhancers [24], and at the histone modification H3K27me3 demarcating polycomb repressed regions [25]. To assess the combinatory impact of these epigenetic modifications in the different metastases on downstream gene expression programs, matched RNA‐seq data were generated providing the most comprehensive epigenome study of mCRPC in a single patient to date.

## 2 Materials and methods

### 2.1 Clinical sample collection

All samples were procured as part of the Johns Hopkins Prostate Cancer Rapid Autopsy Program with approval by the Johns Hopkins Institutional Research Board and written informed consent. The tissue procurement was conducted in accordance with the Declaration of Helsinki. The patient and the patient's next of kin provided consent. Clinicopathological information is described previously [15] and below. Details on genomic profiling have been described previously [15]. In brief, four metastatic samples were taken at the time of autopsy and were compared with the primary tumor using whole‐genome sequencing, copy number variation analysis, immunoprofiling, and targeted sequencing for key drivers.

### 2.2 Patient treatment history

The detailed clinical history of the patient in this study has been reported previously [15]. In brief, the patient was diagnosed age 47 with a Prostate Serum Antigen (PSA) > 40 ng·mL^−1^ and underwent a radical prostatectomy. Five years after surgery, his PSA had risen to > 6 ng·mL^−1^, without any initial radiographic evidence of metastatic disease which prompted the treatment with an investigational prostate cancer vaccine (GVAX) [26]. Over the course of the following 12 years, he experienced disease recurrence in bones, lymph nodes, the prostate bed, skull, lungs, and liver, which were treated with goserelin acetate, bicalutamide, zoledronic acid docetaxel, ^89^Sr, mitoxantrone, abiraterone acetate, etoposide, cisplatin, and external beam radiation along with chemoembolization of the liver lesions. Seventeen years after the initial diagnosis, the patient died of progressive castration‐resistant prostate cancer.

### 2.3 RNA isolation, sequencing, and analysis

RNA was extracted from fresh‐frozen tissue samples using the RNeasy Mini kit (Qiagen, Hilden, Germany). Purified RNA was then used to prepare a sequencing library and sequenced on Applied Biosystems SOLiD (V3). Sequencing reads were aligned to hg18 (NCBI36), and raw counts were generated using Bioscope. We used DESeq2 v1.22.2 [27] to normalize data for library size in R (v3.5.0). All genes that were not expressed across samples (normalized count data < 4) were removed. Subsequently, the data were log transformed. These data were used to plot gene expression heatmaps. To analyze the significant differential gene expression between the two lymph node samples versus two nonlymph node samples, we used DESeq2 with two replicates per condition [27]. The correlation matrix of log transformed normalized data was generated in R using the PerformanceAnalytics package v1.5.3. Gene expression heatmaps were plotted with a heatmap function in the R package NMF v0.21.0 [28] using the log transformed normalized data.

### 2.4 DNA isolation and ChIP‐sequencing

ChIP‐seq for AR, FOXA1, CTCF, H3K27ac, and H3K27me3 were performed using 5 μg of antibody and 50 μL of Protein A/G magnetic beads (Invitrogen, Carlsbad, CA, USA) per sample. Antibodies applied here were AR (Millipore, Burlington, MA, USA, 06‐680, lot#2489005&2943813); FOXA1 (Abcam, Cambridge, UK, ab5089, lot#GR20766‐16); CTCF (Millipore, 07‐729, lot#2887267); H3K27ac (Active Motif, Carlsbad, CA, USA 39133, lot#31814008); H3K27me3 (Sigma‐Aldrich, St. Louis, MO, USA 07‐449, lot#2826067). As control, input of each sample was generated separately. Immunoprecipitated DNA was processed for sequencing using standard protocols and sequenced on an Illumina Hi‐seq 2500 with 65 bp single end reads.

### 2.5 ChIP‐seq analysis

Raw sequencing data were aligned using BWA v0.5.20 to hg19 and filtered for mapping quality > MQ20 with samtools version 1.8 [29]. Duplicate reads were marked with Picard MarkDupes function (version 2.18) (http://broadinstitute.github.io/picard/). We called peaks in all samples for AR, FOXA1, CTCF and H3K27ac against input DNA using two peak callers, macs2 (v2.1.1) [30] and DFilter version 1.5 [31]. The intersect of peaks from both peak callers was used for downstream analysis. Quality of the ChIP‐seq samples for AR, FOXA1, CTCF, and H3K27ac was based on detecting outliers using quantiles of the number of peaks identified in each factor. One sample was removed based on low quality (M40, CTCF, number of peaks = 4250). For H3K27me3, we used the raw signal and did not call peaks. For public data from mCRPC PDX, the input data were not available. To peak call these samples, we used a pooled input of five random mCRPC samples from our own in‐house data. Sample‐shared and sample‐specific site regions and unsupervised clustering analyses of correlation heatmaps were generated using the DiffBind package v2.4.8 in R v3.4.4 with the reads counted in peaks using the dba.count function. To visualize the raw data, heatmaps and profiles were generated with deepTools computeMatrix (v2.0), plotHeatmap, and plotProfile functions [32]. Count per million library normalized data were produced with deepTools bamCoverage and visualized with plotHeatmap [32]. To produce factor specific noise regions for Fig. 3A, for each factor individually, all called peaks were concatenated and bedTools shuffle (v2.26.0) was used to produce a random set of coordinates (not found in the concatenated file) with matching number and length [33]. DNA region and sequence motif enrichment were produced using the CEAS and SeqPos packages, respectively [34, 35]. To subset for H3K27ac co‐occupied AR sample‐specific sites, the sample‐specific AR sites were examined for their respective sample's H3K27ac signal with plotHeatmap *k*‐means function [32]. The top two clusters were taken for further analysis. Gene set over‐representation was examined using the clusterProfiler and DOSE package in R [36] with the MSigDB Hallmarks pathways [37, 38]. Ingenuity Pathway Analysis was used to examine Top Canonical Pathways and Up‐Stream Regulators (Qiagen).

## 3 Results

### 3.1 Epigenomic and transcriptomic profiling of clonal metastases

To determine whether differences in metastatic niche are associated with alterations in the epigenome and transcriptome, we investigated multiple metastasis samples from a single patient, which share a high concordance in driver gene alterations [15]. As described previously [15], multiple metastatic sites were sampled at the time of autopsy, including metastases to the liver (M5_Liver), perigastric lymph node (M38_PLN), lung (M40_Lung), and hilar lymph node (M45_HLN) (Fig. 1A). These samples showed uniformly adenocarcinoma differentiation and were among those previously interrogated at the DNA level using whole‐genome sequencing [15] (Fig. 1A). Genomic analyses revealed a high level of shared driver gene alterations including mutations in *PTEN*, *SPOP*, and *TP53*, copy number alterations including a high‐level copy number gain of the AR locus and structural rearrangements such as an inversion of the *ATRX* locus in all metastases (Fig. 1B). Collectively, these findings establish that from a genomic perspective the lesions from distinct anatomic sites were highly similar (Fig. 1B), as was already reported previously [15].

Minimal intra‐individual genetic heterogeneity allowed us to assess whether diverse metastatic environments would associate with changes in the epigenetic landscape. To investigate the constellation of epigenome and transcriptome changes in each metastatic site, we generated RNA‐seq data and ChIP‐seq data for AR, FOXA1, H3K27ac, and H3K27me3 (Fig. 1C). For ChIP‐seq on AR, FOXA1, CTCF, and H3K27ac, all samples had > 20 million reads per sample on average (Fig. S1A). All samples used in our analyses had at least 10 000 peaks in all factors, which is more than other studies using fresh‐frozen prostate material [9] (Fig. S1B). The very high number of peaks for AR is reflective of the *AR* gene amplification status of these samples [15]. We excluded one CTCF sample based on low number of peaks (M40_Lung, *n* = 4250). Due to technical challenges in calling broad peaks, H3K27me3 data were included only as raw tracks in further analyses.

**Fig. 2:**
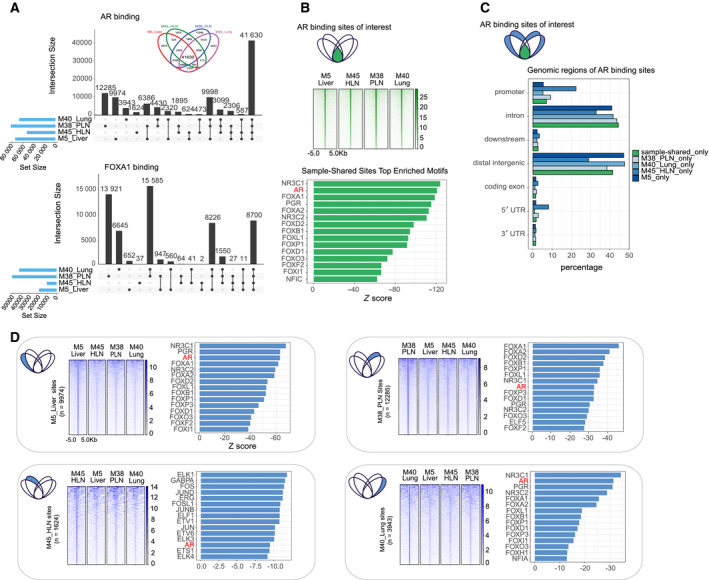
AR and FOXA1 show shared and unique chromatin binding sites. (A) UpSetR diagrams depicting number of shared and unique AR (top) and FOXA1 (bottom) binding sites in four anatomically distinct metastases. Inset for AR (top) shows the same data depicted in Venn diagram format for reference. (B) Top: Schematic indicating the sites visualized in the panel (green: overlapping in all AR samples). Middle: Heatmap showing the average raw AR signal at these sample‐shared sites with 2 kb on either side of the center of the sites. Bottom: Barchart showing *Z* score of the top 15 enriched DNA sequence motifs for sample‐shared sites (AR motif highlighted in red). (C) Top: Schematic indicating the sites visualized in the panel (green: overlapping in all AR samples, blue: sample‐specific AR sites). Bottom: Barchart of the genomic distribution in percentage of AR sample‐specific and sample‐shared peaks. (D) For each panel: Left: Schematic indicating the sites visualized in the panels (blue: sample‐specific AR sites). Middle: Heatmap of raw AR signal in sample‐specific sites with 5 kb on either side of the center of those sites for all samples. Right: Barchart showing *Z* score of top 15 enriched DNA sequence motifs for sites (AR motif highlighted in red).

The AR is a major driver of progression in mCRPC [39, 40]. Between primary prostate cancers of different patients, AR cistromes are highly variable [9]. To determine whether AR binding profiles in different metastatic sites—from within the same patient—are comparably heterogenous, we compared chromatin interactions for AR and its pioneer factor FOXA1 [22] and CTCF using overlapping peaks analysis (Fig. 2A and S2A). We found a large proportion of the AR‐ and FOXA1‐associated regions as shared between samples, with up to 80% of peaks overlapping between samples. This fraction of overlap is similar to that of technical replicates from the same tumor sample [9], indicating a substantial proportion of sites are shared between different metastasis samples from the same patient (Fig. S2A).

The strongest signal for both factors was found where all samples overlapped (the center of the Venn diagram; inset Figs 2A and S2A) representing the shared sample‐shared regions (*n* = 41 630) (Fig. 2B). For AR, we found that shared and sample‐specific binding sites are most frequently enriched for similar genomic locations such as distal intergenic and intronic regions (Fig. 2C) with similar DNA sequence motifs for hormone receptors (AR, NR3C1, PGR) and forkhead motifs (FOXA1, FOXA2) (Fig. 2B: sample‐shared, 2D: sample‐specific, Table S1). As expected, we found AR and FOXA1 motifs to be significantly enriched in all sample‐specific binding sites; however, they were not always found among the top most significant motifs (Fig. 2D, Table S1). These findings suggest a similar coordinated cistrome between metastatic sites. Interestingly, for the AR sites selectively bound in sample M45_HLN (representing 3% of the total peaks in this sample), we identified motif enrichment for additional transcription factors such as ELK1 (ETS transcription factor family), FOS, and JUN, suggesting some level of selective transcription factor functionality in this metastatic sample (Fig. 2D, bottom left, Table S1). Of note, M45_HLN showed focal areas of necrosis on H&E which might provide a histomorphological correlate to the cistromic differences. As expected, for all samples analyzed, AR binding signal intensity at sample‐specific sites was stronger in the respective sample (Fig. 2D). Cumulatively, our findings indicate a striking homogeneity in the cistrome centering on AR and FOXA1 among the four organ site metastases at the end stage of the patient's clinical course.

### 3.2 Sample‐shared epigenetic regions have similar patterns associated with the potential for transcriptional activity

**Fig. 3:**
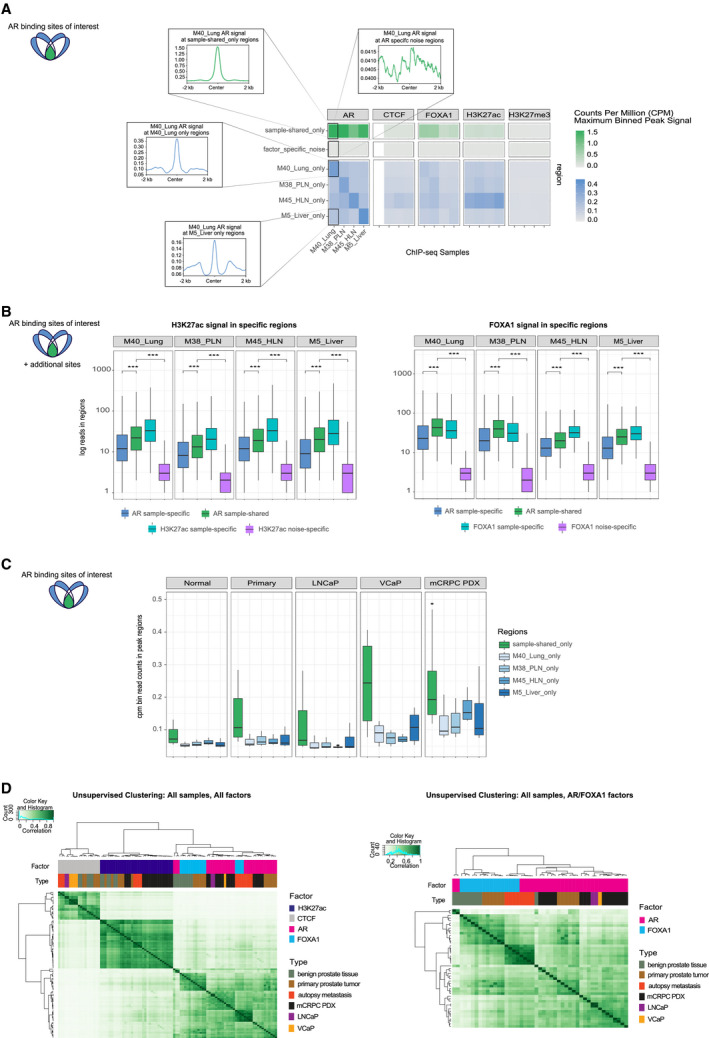
Multicistromics data integration between metastatic sites reveals metastatic disease specific epigenetic patterns and prostate lineage programming. (A) Left: Schematic indicating the sites visualized in the panel (green: overlapping in all AR samples, blue: sample‐specific AR sites). Right (top, green): Heatmap of sample‐shared‐only signal in all factors, AR, CTCF, FOXA2, H3K27ac, and H3K27me3. Scale bar indicates counts per million (CPM) maximum binned signal at peak regions (0–1.5). Right (bottom, green): Heatmap of factor_specific_noise signal in factors, AR, CTCF, FOXA2, H3K27ac, and H3K27me3. Scale bar indicates CPM maximum binned signal at peak regions (0–1.5). Insets show the profile plot of signal from AR data from M40_Lung for sample‐shared_only (top left) and AR factor specific noise (top right) regions. Right (blue): Heatmap of sample‐specific regions for all ChIP‐seq data in all factors. Scale bar indicates CPM maximum binned signal at peak regions (0–0.45). Insets show the profile plot of signal from of AR data from M40_Lung for M40_Lung_only sample‐specific regions (middle left) and M5_Liver_only sample‐specific (bottom left) regions. (B) Left: Boxplots (for all: the line represents median, bottom, and top of boxes show the 25th and 75th percentile (interquartile range), vertical line represents 1.5 times the interquartile range) indicating the log read counts in H3K27ac data for AR sample‐specific (blue; *n* = 67 289, 82 355, 53 248, and 74 895, left to right), AR sample‐shared (green; *n* = 41 630 for all samples) H3K27ac sample‐specific (turquoise; *n* = 49 375, 44 051, 37 514, and 55 173, left to right) and H3K27ac noise‐specific (purple; *n* = 463 900 for all samples) regions. Right: Boxplots indicating the log read counts in FOXA1 data for AR sample‐specific (blue; *n* is same as left panel), AR sample‐shared (green; *n* is same as left panel) FOXA1 sample‐specific (turquoise; *n* = 41 576, 49 246, 10 542, and 19 413, left to right) and FOXA1 noise‐specific (purple; *n* = 120 777 for all samples) regions [*** indicates *P* < 0.005 (Wilcoxon test)]. (C) Left: Schematic indicating the sites visualized in the panel (green: overlapping in all AR samples, blue: sample‐specific AR sites). Right: Boxplots of binned read CPM data (*n* of bins = 100) at peaks for sample‐shared‐only and sample‐specific regions for benign prostate tissue (Normal), primary prostate tumor (Primary) and LNCaP and VCaP cell line samples and mCRPC PDX samples. (D) Left: Unsupervised clustering of correlation heatmap using read count data in peaks for benign prostate tissue (dark green), primary prostate tumor (brown), autopsy metastasis (orange), mCRPC patient‐derived (PDX) samples (black), and LNCaP (purple) and VCaP (yellow) samples for AR (pink), FOXA1 (turquoise), CTCF (gray), and H3K27ac (dark blue) data. Pearson correlation coefficient shown from 0 (white) to 1 (dark green). Hierarchical clustering was performed with the complete linkage method. Right: Unsupervised clustering of correlation heatmap using read count data in peaks of AR (pink) and FOXA1 (turquoise) data only for benign prostate tissue (dark green), primary prostate tumor (brown), autopsy metastasis (orange), mCRPC patient‐derived (PDX) samples (black), and LNCaP (purple) and VCaP (yellow) cell lines. Pearson correlation coefficient shown from 0 (white) to 1 (dark green). Hierarchical clustering was performed as in Left panel.

In order to better characterize the epigenomic landscape and understand the underlying regulatory potential, we overlaid the AR binding regions (sample‐shared and sample‐specific) with the other datastreams for each sample. This allowed us to uncover epigenetic patterns in both sample‐shared and sample‐specific regions. In sample‐shared AR regions, we found high H3K27ac signal, medium CTCF, low H3K27me3 signal, and high FOXA1 signal, suggesting that these highly conserved AR‐bound regions are found in active enhancers/promoters (Fig. 3A, sample‐shared_only). Furthermore, for all factor data we also included signal at a random set of regions (factor_specific_noise), which shows very low signal in ‘noise’ regions for all factors and samples indicating the quality of signal strength in sample‐shared and sample‐specific regions (Fig. 3A, factor_specific_noise; see methods). We hypothesized that AR sites, which are absent in a particular sample (i.e., found in sample‐specific regions), would also be devoid of enhancer activity in that specific sample. To test this, we also examined the H3K27ac data in AR sample‐specific regions and identified no discernable sample‐specific pattern as observed for AR. In addition, in AR sample‐specific sites, there was no overall increase in H3K27me3. Together, these data support the conclusion that absence or presence of AR at a particular site is not associated with differential enhancer activity (Fig. 3A, sample_specific_regions). To further confirm this, we examined the number of reads in sample‐shared and sample‐specific AR regions in H3K27ac data for each sample. We observed that H3K27ac signal in sample‐specific regions remains present, as indicated by signal significantly higher than factor‐specific noise (Wilcoxon test, *P* < 0.005), albeit significantly lower than in sample‐shared regions (Fig. 3B, Wilcoxon test, *P* < 0.005). Moreover, we observed the same pattern for FOXA1, further supporting the hypothesis that absent AR sites are not devoid of enhancer activity (Fig. 3B, Wilcoxon test, *P* < 0.005). Although the number of reads is of sufficient depth for calling quality peaks (average mean depth > 100), the number of reads in AR sample‐specific regions for ChIP‐seq datasets of AR, H3K27ac, and FOXA1 is consistently lower than sample‐shared regions (Figs 2D and 3A,B). Interestingly, the fact that FOXA1 does not follow a strong sample‐specific pattern similar to AR binding suggests that there is an AR‐independent function of FOXA1 in these metastases samples, in line with recent evidence in primary tumor development [41]. Cumulatively, these data suggest that when AR is absent at a specific genomic region in a sample‐specific manner, that enhancer remains active.

The shared AR binding sites (sample‐shared) are very highly bound by H3K27ac and FOXA1. Based on these findings, we hypothesized that the shared AR binding sites represent core binding events of a prostate lineage‐specific epigenetic program that is established during organogenesis and maintained throughout cancer progression. To test this, we investigated the distribution of AR sites in previously published ChIP‐seq datasets from benign prostate tissues, primary prostate cancers [10] and cell lines (LNCaP and VCaP [42, 43] and in mCRPC patient‐derived xenografts (PDX) [18]. Importantly, we found a consistent significantly stronger enrichment of AR signal at the respective sample‐shared epigenetic sites in benign prostate tissues, primary tumors, cell lines [42, 43], and mCRPC PDX samples [18] compared to the sample‐specific sites (Fig. 3C) (Wilcoxon test, *P*‐values < 0.005).

To formally test the hypothesis that sample‐shared AR regions are associated with prostate cell survival, we evaluated the enrichment of essential genes (LNCaP) in the gene list that was associated with H3K27ac‐positive (active) sample‐shared AR binding sites [44]. Essential genes in LNCaP cells (FDR < 0.05) were found to be significantly enriched in genes associated with the active sample‐shared AR sites as defined by proximity (within 20 kb of TSS) (hypergeometric test, *P* = 0.03, Fig. S3A). This demonstrates these sample‐shared regions are likely to be essential and associated with genes for prostate cancer cell proliferation.

To better understand the relationship between all factors and sample types, we examined the correlation of samples using peak occupancy (reads in peaks) and unsupervised clustering. We found sample clustering to be driven by factor, with CTCF and H3K27ac data clustering separately from AR/FOXA1 data, regardless of sample type (Fig. 3D). This reinforces our earlier observation that H3K27ac did not show the same signal of the AR/FOXA1 binding in sample‐specific regions. As the AR and FOXA1 data were intermingled based on initial unsupervised clustering, we next focused exclusively on AR/FOXA1 datasets for all sample types in the same manner (Fig. 3D). This analysis was aimed to determine underlying relationships within AR/FOXA1 data alone. Here, we identified the AR and FOXA1 autopsy metastasis samples our autopsy series cluster differently from other samples including mCRPC PDX models [18], implying a specific epigenetic pattern selectively found in this individual patient with metastatic disease, which is highly conserved between different metastatic sites.

### 3.3 Genes connected to active differential AR‐bound sites do not show altered transcriptional output

**Fig. 4:**
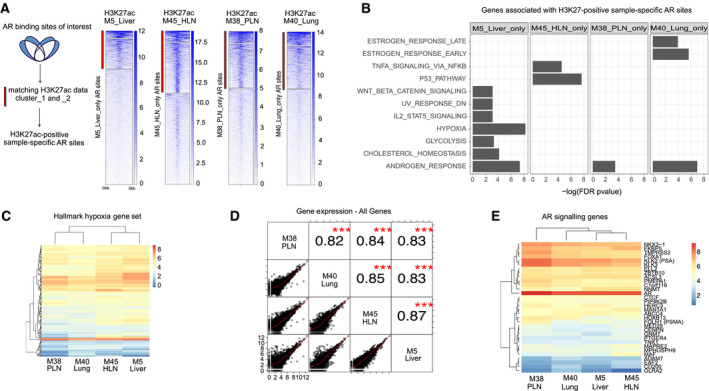
Cistromic differences have minimal impact on transcriptional output in anatomically distinct metastatic sites. (A) Left, Schematic of subset analysis approach. Sample enriched AR sites (blue) were subsetted for matching H3K27ac signal (*k*‐means cluster 1 and 2, red bar). Right: For each sample, sample‐specific AR sites were examined for raw H3K27ac signal (red bar indicates sites used to associate with nearest gene). (B) Barchart of −log_10_(FDR) enrichment for Hallmarks Gene Sets (MSigDB) in sample‐specific gene sets. Missing bar indicates the gene set was not among the top 12 significantly enriched sets in that sample. (C) Heatmap of gene expression for Hallmark Hypoxia Gene Set genes. Normalized gene expression shown from low (blue) to high (red). (D) Correlation matrix of normalized log‐transformed gene expression data for all samples for all genes. Pearson correlation and significance are indicated in the top right (*** indicates *P* < 0.0001). (E) Heatmap of normalized gene expression for of AR signaling genes (27) (top) and AR, FOXA1, and HOXB13 genes (bottom). Normalized gene expression shown from low (blue) to high (red).

To better understand the metastatic site‐selective AR regions and their potential impact on downstream transcription, we analyzed gene expression for these regions. As active enhancers and promoters are associated with H3K27ac, for each sample we focused on AR sample‐specific regions which are co‐occupied with H3K27ac (matching sample) using *k*‐means clustering (Fig. 4A). Subsequently, H3K27ac‐positive AR sites for each metastatic site were analyzed for their impact on gene expression programs, by identifying the closest gene (within 20 kb) for each site (Fig. 4A, Table S2). Genes identified for each sample in this manner were tested for their enrichment in Hallmarks Gene Sets, using the hypergeometric test. No enriched gene sets were found that overlapped between metastatic sites with the exception of Androgen Response found in three samples (Fig. 4B). These samples also showed on average the highest percentage of sample‐specific genes overlapping in the Androgen Response gene set (Fig. S4A). Very few genes from the Androgen Response gene set overlap in the other enriched gene sets (Fig. S4B). Ingenuity Pathway Analysis also revealed Molecular Mechanisms of Cancer as a Top Canonical Pathway among samples as well as Up‐Stream Regulators including beta‐estradiol, NR3C1 (GR) TP53 and Estrogen Receptor 1 (Table S2). Surprisingly, while hypoxia was the most sample‐specific enriched Hallmark gene set based on ChIP‐seq data integration (enriched in M5_Liver_only), we observed very stable gene expression for the genes in this gene set across samples (Fig. 4C). We also saw highly correlated expression across all significantly enriched Hallmark gene sets for all samples (Fig. S4C). This indicates that although specific gene sets may be enriched in specific samples based on epigenetic marks, it is not translated to alterations in expression of the corresponding genes in the gene set.

Much to our surprise, homogeneity was found overall; very few differential pathways were found associated with the sample‐specific differentially bound active AR sites. Therefore, we next investigated the correlation of normalized gene expression for all genes across samples. Also, in this analysis, despite the observed selectivity of AR sites between metastatic sites, gene expression for all samples was highly significantly correlated (Fig. 4D). To further confirm our results, we investigated known AR signaling genes [8] more closely and as well as genes encoding for proteins that are most commonly associated with AR biology in prostate cancer (FOXA1, AR, and HOXB13), and found them virtually identical across all samples (Fig. 4E). Further corroborating this, we formally tested for differential gene expression between lymph node‐derived samples (M38_PLN and M45_HLN) and organ derived samples (M5_Liver and M40_Lung). We found only two genes (SLC34A2 and DMBT1) as significantly differentially expressed between these groups (FDR ≤ 0.05 and absolute (logFC ≥ 2). Together, these data show that expression of genes with metastatic site‐selective AR/H3K27ac sites is remarkably similar. These findings suggest a robustness of the transcriptional output despite differences in transcriptional regulation between different metastatic sites.

## 4 Discussion

Tumor‐stromal interactions are widely accepted to contribute to the tumorigenic and metastatic process, but a direct connection between the tumor metastatic niche and tumor epigenome remains thus far elusive. Here, we have profiled clonal metastases from four distinct metastatic sites of a patient who died of mCRPC. We applied a comprehensive integrated approach to capture both transcriptomic and epigenomic alterations, in an effort to better understand the heterogeneity of the epigenome and cistromic changes between different metastatic sites. The unique strength of this study lies in the assessment of multiple mCRPC metastatic lesions from different sites that were derived from a single patient, which show a very high level of genomic homogeneity. This design allowed us to interrogate epigenomic differences in the background of a shared driver gene alterations.

While the vast majority of AR chromatin binding sites were conserved among all metastatic sites, we identified a subset of AR‐bound regions that were sample/metastatic site‐specific. These regions were similar, both in genomic region as well as in DNA sequence motif preference, with the exception of one sample, M45_HLN that shows focal areas of necrosis that may explain cistromic differences. FOXA1 facilitates but does not have transactivating potential alone. We hypothesize differential FOXA1 does not necessarily translate to differences in downstream transcriptomics depending on the transcription factor, co‐factor binding and regulatory effects. In a subset analysis of differential AR‐bound regions marked by H3K27ac, we found that there were very few differences in gene expression programs that are associated with differentially bound regions. This indicates that although there were differences in active AR‐bound regulatory elements, these changes did not translate into differential transcriptional outputs. Importantly, we did not observe sample‐selectivity in H3K27ac occupancy at the AR‐selective sites but did still observe H3K27ac binding, indicating the absence of AR binding does not impact the activity of the corresponding enhancer region. In addition, we did not observe an increase in H3K27me3 levels when AR is absent from a specific site, supporting the conclusion that enhancer activity remains in the absence of AR. However, as both FOXA1 and H3K27ac were present in all samples at specific regions where AR was sample‐specific, we hypothesize that possible other yet‐to‐be classified transcription factors may compensate for AR at these regions. It is also possible that at the sample‐specific AR sites, the AR dynamics in that particular sample at that site were less transient. Interestingly, we found binding at core AR regions is stronger in all sample types: normal, primary tumors, cell lines, and PDX samples, indicating an early epigenetic programming event. Of note, we also found zero overlap of core AR regions with metastasis‐specific AR sites [18] further showing that our core AR sites that were also identified in healthy tissue and primary prostate cancers are separate biologically from metastasis‐specific AR sites. We note the variation of the PDX samples is higher than other sample types, which may be explained by the many different locations these samples were derived from including lymph nodes, bladder, liver, femur, Transurethral Resection of the Prostate, and cells from ascites [45]. Cell lines consistently showed a different pattern for binding data from the autopsy metastasis samples, revealing the inadequacy of current model systems to recapitulate the complexity of metastatic samples. The metastases studied here were all from soft tissues (lung, liver, lymph nodes), and it could be argued that the most common metastatic site for prostate cancer, the bone, may have a substantially different micro‐environment to significantly modulate the epi‐transcriptome. This is worth investigating further in future studies, although unfortunately, clinical samples of sufficient quality for molecular profiling are difficult to obtain from bone. We also found that the global transcriptomic signals from each sample are highly correlated, which has been previously shown [12]. With the addition of ChIP‐seq data for multiple transcription factors and epigenetic marks as presented in our study, we were able to position this transcriptomic stability in the context of a differential epigenome. It should be noted that we did find a high proportion of AR/FOXA1 sites that are shared between all samples, which may indicate an overall dominant clonal transcriptional survival program beyond the sample‐specific sites. Taken together, these findings suggest that despite tumor site‐specific epigenome heterogeneity, there is a remarkable robustness of core transcriptional clonal survival programs that are shared between all metastases in a given patient indicating a minimal impact of sampling bias in research focused on mCRPC disease. These findings are in line with previous results showing DNA methylation alterations that varied between metastatic sites within a single patient had little impact on *cis* gene expression [46].

The patient in this study was first diagnosed with distant metastatic disease (bone, lymph nodes) around 13 years after radical prostatectomy. In the following years, up until death, he developed additional metastases with extensive involvement of the liver, lung and lymph nodes [15]. Over the course of that time, many factors could have been predicted to play a large role in generating heterogeneous transcriptional programs at different metastatic sites, such as tumor evolution and clonal resistance to multiple systemic therapies as well as (epi)genetic drift, yet we observe no evidence of this. Rather, a conserved core clonal survival transcriptional program was manifested and preserved.

The biological implications of these findings should be interpreted with caution, since there are several limitations to this study. The study is based on the in‐depth analysis of a single case, which raises concerns about the generalizability of the findings. However, the value of ‘*N* = 1’ studies is increasingly recognized [47], since insights from the integrated analyses of the patient’s clinical history and molecular findings can often get diluted when analyzing larger case series. Therefore, we hope that this report that takes advantage of the comprehensive clinical and molecular annotations available for this case will stimulate future studies in the field.

It is important to note that all samples analyzed here were procured at an autopsy and are therefore representative of metastatic tumor cell populations that have evolved in this case over a period of over 17 years and have developed resistance to numerous therapies. It is therefore possible that the tumor burden at autopsy appears from a genetic and epigenetic standpoint more homogeneous, since tumor cells had to pass through numerous treatment‐induced clonal bottle necks. It remains to be explored if samples collected in a longitudinal fashion at different stages of tumor progression show a similarly conserved core epigenome program. Although additional longitudinally collected samples were available for this case, they comprise > 25‐year‐old Formalin‐Fixed Paraffin‐Embedded (FFPE) material, which is not compatible with the ChIP‐seq protocols used in this study. This highlights the need for thoughtful prospective sample collection and for the development of robust protocols to assess cistromic characterization in FFPE materials.

This case is characterized by high expression of prostate specific markers and no evidence of divergent differentiation to neuroendocrine (NE) or AR negative prostate cancer [15, 48, 49, 50, 51, 52]. Although this tumor phenotype (i.e., high AR expression, absence of NE marker expression) still represents the most commonly observed molecular subtype of mCRPC in contemporary rapid autopsy cohorts [49, 53], the emergence of treatment‐induced phenotypes that are characterized by NE marker expression and/or absence of AR expression need to be carefully evaluated [48]. Indeed, a more recent study provided first evidence for major DNA methylation changes associated with NE features [54]. Tumors with evidence of divergent differentiation are most likely to harbor profound epigenetic changes that are associated with distinct tumor cell phenotypes. Therefore, additional studies are needed to address the role of epigenome changes in treatment associated phenotypic plasticity.

## 5 Conclusions

In summary, this study represents the first in field evaluation of cistromic and epigenomic heterogeneity in any type of metastatic cancer and sets the stage for further studies in this field which will likely yield major new insights into the biology of advanced prostate cancer.

## Conflict of interest

LW has received funding from Genmab, BV. WGN is on the Scientific Advisory Board of Cepheid, Inc. WZ has received research funding and speaker’s compensation from Astellas Pharma.

## Author contributions

YZ and SY performed the experiments. TMS performed computational analyses. AMD‐M, TMS, MCH, and WZ conceived study design. LW, AMB, MCH, and WZ performed study supervision. TJ, AMD‐M, and YZ performed sample procurement and processing. SY and WGN provided infrastructural support. TMS, MCH, and WZ wrote manuscript, with critical input from MLF and JWS.

### Peer Review

The peer review history for this article is available at https://publons.com/publon/10.1002/1878‐0261.12923.

## Supporting information


**Fig. S1.** ChIP‐seq quality measures of anatomically distinct metastases. (A) Boxplots of millions of mapped reads > MQ20 for AR (pink), FOXA1 (turquoise), CTCF (gray) and H3K27ac (dark blue). AR, FOXA1 and H3K27ac have *n* = 4. CTCF has *n* = 3. The one outlier H3K27ac sample (outlined in red) indicates the data are less than the 25th percentile (Q1) ‐ 1.5*(interquartile range). (B) Boxplots of number of peaks of AR (pink), FOXA1 (turquoise), CTCF (gray) and H3K27ac (dark blue). Number of samples is the same as left plot.

**Fig. S2.** Shared and unique chromatin binding sites of AR and FOXA1. (A) Venn diagrams depicting the number of shared and unique AR (left), FOXA1 (center) and CTCF (right) binding sites in 3 ‐ 4 anatomically distinct metastases.

**Fig. S3.** Overlap of LNCaP essential genes and genes associated with active sample‐shared AR sites. (A) UpSetR diagram depicting number of shared (red) and unique (gray) genes in LNCaP essential genes [47] and genes associated with active (H3K27ac‐positive) sample‐shared AR sites.

**Fig. S4.** Percentage and overlap of genes in significantly enriched Hallmarks gene sets and gene set expression correlation. (A) Stacked barplots indicating the percentage of genes which were in sample‐specific lists (orange) in significantly enriched gene sets (gray). (B) UpSetR diagram depicting the shared and unique genes in Hallmarks gene sets that were sample‐specifically significantly enriched. Red box indicates overlaps of genes in gene sets with the Androgen Response gene set. (C) Correlation matrix of normalized log‐transformed gene expression data for remaining Hallmark gene sets for all samples. Pearson correlation and significance are indicated in the top right (*** indicates *P* < 0.0001).

**Table S1.** SeqPos DNA motif enrichment at AR sites (shared and sample‐specific).

**Table S2.** Genes associated with H3K27ac‐positive sample selective AR sites and their respective Ingenuity Pathway Analysis Top Canonical Pathways and Upstream Regulators.

## Data Availability

ChIP‐seq and RNA‐seq data are deposited in GEO under the SuperSeries GSE152231.

## References

[1] Hu R , Dunn TA , Wei S , Isharwal S , Veltri RW , Humphreys E , Han M , Partin AW , Vessella RL , Isaacs WB *et al*. (2009) Ligand‐independent androgen receptor variants derived from splicing of cryptic exons signify hormone‐refractory prostate cancer. Cancer Res 69, 16–22. 19117982 10.1158/0008-5472.CAN-08-2764 PMC2614301

[2] Suzuki H , Sato N , Watabe Y , Masai M , Seino S & Shimazaki J (1993) Androgen receptor gene mutations in human prostate cancer. J Steroid Biochem Mol Biol 46, 759–765. 8274409 10.1016/0960-0760(93)90316-o

[3] Taplin ME , Bubley GJ , Shuster TD , Frantz ME , Spooner AE , Ogata GK , Keer HN & Balk SP (1995) Mutation of the androgen‐receptor gene in metastatic androgen‐independent prostate cancer. N Engl J Med 332, 1393–1398. 7723794 10.1056/NEJM199505253322101

[4] Taplin ME , Bubley GJ , Ko YJ , Small EJ , Upton M , Rajeshkumar B & Balk SP (1999) Selection for androgen receptor mutations in prostate cancers treated with androgen antagonist. Cancer Res 59, 2511–2515. 10363963

[5] Robinson D , Van Allen EM , Wu Y‐M , Schultz N , Lonigro RJ , Mosquera J‐M , Montgomery B , Taplin M‐E , Pritchard CC , Attard G *et al*. (2015) Integrative clinical genomics of advanced prostate cancer. Cell 162, 454. 28843286 10.1016/j.cell.2015.06.053

[6] Visakorpi T , Hyytinen E , Koivisto P , Tanner M , Keinänen R , Palmberg C , Palotie A , Tammela T , Isola J & Kallioniemi OP (1995) *In vivo* amplification of the androgen receptor gene and progression of human prostate cancer. Nat Genet 9, 401–406. 7795646 10.1038/ng0495-401

[7] Takeda DY , Spisák S , Seo J‐H , Bell C , O’Connor E , Korthauer K , Ribli D , Csabai I , Solymosi N , Szállási Z *et al*. (2018) A somatically acquired enhancer of the androgen receptor is a noncoding driver in advanced prostate cancer. Cell 174, 422–432.e13. 29909987 10.1016/j.cell.2018.05.037 PMC6046260

[8] Stelloo S , Nevedomskaya E , van der Poel HG , de Jong J , van Leenders GJLH , Jenster G , Wessels LFA , Bergman AM & Zwart W (2015) Androgen receptor profiling predicts prostate cancer outcome. EMBO Mol Med 7, 1450–1464. 26412853 10.15252/emmm.201505424 PMC4644377

[9] Stelloo S , Nevedomskaya E , Kim Y , Schuurman K , Valle‐Encinas E , Lobo J , Krijgsman O , Peeper DS , Chang SL , Feng FY‐C *et al*. (2018) Integrative epigenetic taxonomy of primary prostate cancer. Nat Commun 9, 4900. 30464211 10.1038/s41467-018-07270-2 PMC6249266

[10] Mazrooei P , Kron KJ , Zhu Y , Zhou S , Grillo G , Mehdi T , Ahmed M , Severson TM , Guilhamon P , Armstrong NS *et al*. (2019) Cistrome partitioning reveals convergence of somatic mutations and risk variants on master transcription regulators in primary prostate tumors. Cancer Cell 36, 674–689.e6. 31735626 10.1016/j.ccell.2019.10.005

[11] Pomerantz MM , Li F , Takeda DY , Lenci R , Chonkar A , Chabot M , Cejas P , Vazquez F , Cook J , Shivdasani RA *et al*. (2015) The androgen receptor cistrome is extensively reprogrammed in human prostate tumorigenesis. Nat Genet 47, 1346–1351. 26457646 10.1038/ng.3419 PMC4707683

[12] Kumar A , Coleman I , Morrissey C , Zhang X , True LD , Gulati R , Etzioni R , Bolouri H , Montgomery B , White T *et al*. (2016) Substantial interindividual and limited intraindividual genomic diversity among tumors from men with metastatic prostate cancer. Nat Med 22, 369–378. 26928463 10.1038/nm.4053 PMC5045679

[13] Gundem G , Van Loo P , Kremeyer B , Alexandrov LB , Tubio JMC , Papaemmanuil E , Brewer DS , Kallio HML , Högnäs G , Annala M *et al*. (2015) The evolutionary history of lethal metastatic prostate cancer. Nature 520, 353–357. 25830880 10.1038/nature14347 PMC4413032

[14] Liu W , Laitinen S , Khan S , Vihinen M , Kowalski J , Yu G , Chen L , Ewing CM , Eisenberger MA , Carducci MA *et al*. (2009) Copy number analysis indicates monoclonal origin of lethal metastatic prostate cancer. Nat Med 15, 559–565. 19363497 10.1038/nm.1944 PMC2839160

[15] Haffner MC , Mosbruger T , Esopi DM , Fedor H , Heaphy CM , Walker DA , Adejola N , Gürel M , Hicks J , Meeker AK *et al*. (2013) Tracking the clonal origin of lethal prostate cancer. J Clin Invest 123, 4918–4922. 24135135 10.1172/JCI70354 PMC3809798

[16] Grasso CS , Wu Y‐M , Robinson DR , Cao X , Dhanasekaran SM , Khan AP , Quist MJ , Jing X , Lonigro RJ , Brenner JC *et al*. (2012) The mutational landscape of lethal castration‐resistant prostate cancer. Nature 487, 239–243. 22722839 10.1038/nature11125 PMC3396711

[17] Hong MKH , Macintyre G , Wedge DC , Van Loo P , Patel K , Lunke S , Alexandrov LB , Sloggett C , Cmero M , Marass F *et al*. (2015) Tracking the origins and drivers of subclonal metastatic expansion in prostate cancer. Nat Commun 6, 6605. 25827447 10.1038/ncomms7605 PMC4396364

[18] Pomerantz MM , Qiu X , Zhu Y , Takeda DY , Pan W , Baca SC , Gusev A , Korthauer KD , Severson TM , Ha G *et al*. (2020) Prostate cancer reactivates developmental epigenomic programs during metastatic progression. Nat Genet 52, 790–799. 32690948 10.1038/s41588-020-0664-8 PMC10007911

[19] Ferrari AC , Alumkal JJ , Stein MN , Taplin M‐E , Babb J , Barnett ES , Gomez‐Pinillos A , Liu X , Moore D , DiPaola R *et al*. (2019) Epigenetic therapy with panobinostat combined with bicalutamide rechallenge in castration‐resistant prostate cancer. Clin Cancer Res 25, 52–63. 30224345 10.1158/1078-0432.CCR-18-1589

[20] Quigley DA , Dang HX , Zhao SG , Lloyd P , Aggarwal R , Alumkal JJ , Foye A , Kothari V , Perry MD , Bailey AM *et al*. (2018) Genomic hallmarks and structural variation in metastatic prostate cancer. Cell 175, 889. 30340047 10.1016/j.cell.2018.10.019

[21] van Dessel LF , van Riet J , Smits M , Zhu Y , Hamberg P , van der Heijden MS , Bergman AM , van Oort IM , de Wit R , Voest EE *et al*. (2019) The genomic landscape of metastatic castration‐resistant prostate cancers reveals multiple distinct genotypes with potential clinical impact. Nat Commun 10, 5251. 31748536 10.1038/s41467-019-13084-7 PMC6868175

[22] Robinson JLL & Carroll JS (2012) FoxA1 is a key mediator of hormonal response in breast and prostate cancer. Front Endocrinol 3, 68. 10.3389/fendo.2012.00068 PMC3355944 22649425

[23] Phillips JE & Corces VG (2009) CTCF: master weaver of the genome. Cell 137, 1194–1211. 19563753 10.1016/j.cell.2009.06.001 PMC3040116

[24] Creyghton MP , Cheng AW , Welstead GG , Kooistra T , Carey BW , Steine EJ , Hanna J , Lodato MA , Frampton GM , Sharp PA *et al*. (2010) Histone H3K27ac separates active from poised enhancers and predicts developmental state. Proc Natl Acad Sci USA 107, 21931–21936. 21106759 10.1073/pnas.1016071107 PMC3003124

[25] Fischle W , Wang Y , Jacobs SA , Kim Y , Allis CD & Khorasanizadeh S (2003) Molecular basis for the discrimination of repressive methyl‐lysine marks in histone H3 by Polycomb and HP1 chromodomains. Genes Dev 17, 1870–1881. 12897054 10.1101/gad.1110503 PMC196235

[26] Simons JW , Mikhak B , Chang JF , DeMarzo AM , Carducci MA , Lim M , Weber CE , Baccala AA , Goemann MA , Clift SM *et al*. (1999) Induction of immunity to prostate cancer antigens: results of a clinical trial of vaccination with irradiated autologous prostate tumor cells engineered to secrete granulocyte‐macrophage colony‐stimulating factor using ex vivo gene transfer. Cancer Res 59, 5160–5168. 10537292

[27] Love MI , Huber W & Anders S (2014) Moderated estimation of fold change and dispersion for RNA‐seq data with DESeq2. Genome Biol 15, 550. 25516281 10.1186/s13059-014-0550-8 PMC4302049

[28] Gaujoux R & Seoighe C (2010) A flexible R package for nonnegative matrix factorization. BMC Bioinformatics 11, 367. 20598126 10.1186/1471-2105-11-367 PMC2912887

[29] Li H , Handsaker B , Wysoker A , Fennell T , Ruan J , Homer N , Marth G , Abecasis G . Durbin R & 1000 Genome Project Data Processing Subgroup (2009) The Sequence Alignment/Map format and SAMtools. Bioinformatics 25, 2078–2079. 19505943 10.1093/bioinformatics/btp352 PMC2723002

[30] Zhang Y , Liu T , Meyer CA , Eeckhoute J , Johnson DS , Bernstein BE , Nusbaum C , Myers RM , Brown M , Li W *et al*. (2008) Model‐based analysis of ChIP‐Seq (MACS). Genome Biol 9, R137. 18798982 10.1186/gb-2008-9-9-r137 PMC2592715

[31] Kumar V , Muratani M , Rayan NA , Kraus P , Lufkin T , Ng HH & Prabhakar S (2013) Uniform, optimal signal processing of mapped deep‐sequencing data. Nat Biotechnol 31, 615–622. 23770639 10.1038/nbt.2596

[32] Ramírez F , Ryan DP , Grüning B , Bhardwaj V , Kilpert F , Richter AS , Heyne S , Dündar F & Manke T (2016) deepTools2: a next generation web server for deep‐sequencing data analysis. Nucleic Acids Res 44, W160–165. 27079975 10.1093/nar/gkw257 PMC4987876

[33] Quinlan AR & Hall IM (2010) BEDTools: a flexible suite of utilities for comparing genomic features. Bioinformatics 26, 841–842. 20110278 10.1093/bioinformatics/btq033 PMC2832824

[34] Liu T , Ortiz JA , Taing L , Meyer CA , Lee B , Zhang Y , Shin H , Wong SS , Ma J , Lei Y *et al*. (2011) Cistrome: an integrative platform for transcriptional regulation studies. Genome Biol 12, R83. 21859476 10.1186/gb-2011-12-8-r83 PMC3245621

[35] Shin H , Liu T , Manrai AK & Liu XS (2009) CEAS: cis‐regulatory element annotation system. Bioinformatics 25, 2605–2606. 19689956 10.1093/bioinformatics/btp479

[36] Yu G , Wang L‐G , Yan G‐R & He Q‐Y (2015) DOSE: an R/Bioconductor package for disease ontology semantic and enrichment analysis. Bioinformatics 31, 608–609. 25677125 10.1093/bioinformatics/btu684

[37] Liberzon A , Birger C , Thorvaldsdóttir H , Ghandi M , Mesirov JP & Tamayo P (2015) The Molecular Signatures Database (MSigDB) hallmark gene set collection. Cell Syst 1, 417–425. 26771021 10.1016/j.cels.2015.12.004 PMC4707969

[38] Subramanian A , Tamayo P , Mootha VK , Mukherjee S , Ebert BL , Gillette MA , Paulovich A , Pomeroy SL , Golub TR , Lander ES *et al*. (2005) Gene set enrichment analysis: a knowledge‐based approach for interpreting genome‐wide expression profiles. Proc Natl Acad Sci USA 102, 15545–15550. 16199517 10.1073/pnas.0506580102 PMC1239896

[39] Chen CD , Welsbie DS , Tran C , Baek SH , Chen R , Vessella R , Rosenfeld MG & Sawyers CL (2004) Molecular determinants of resistance to antiandrogen therapy. Nat Med 10, 33–39. 14702632 10.1038/nm972

[40] Holzbeierlein J , Lal P , LaTulippe E , Smith A , Satagopan J , Zhang L , Ryan C , Smith S , Scher H , Scardino P *et al*. (2004) Gene expression analysis of human prostate carcinoma during hormonal therapy identifies androgen‐responsive genes and mechanisms of therapy resistance. Am J Pathol 164, 217–227. 14695335 10.1016/S0002-9440(10)63112-4 PMC1602218

[41] Zhou S , Hawley JR , Soares F , Grillo G , Teng M , Madani Tonekaboni SA , Hua JT , Kron KJ , Mazrooei P , Ahmed M *et al*. (2020) Noncoding mutations target cis‐regulatory elements of the FOXA1 plexus in prostate cancer. Nat Commun 11, 441. 31974375 10.1038/s41467-020-14318-9 PMC6978390

[42] Malinen M , Niskanen EA , Kaikkonen MU & Palvimo JJ (2017) Crosstalk between androgen and pro‐inflammatory signaling remodels androgen receptor and NF‐κB cistrome to reprogram the prostate cancer cell transcriptome. Nucleic Acids Res 45, 619–630. 27672034 10.1093/nar/gkw855 PMC5314794

[43] Takayama K‐I , Suzuki T , Fujimura T , Urano T , Takahashi S , Homma Y & Inoue S (2014) CtBP2 modulates the androgen receptor to promote prostate cancer progression. Cancer Res 74, 6542–6553. 25228652 10.1158/0008-5472.CAN-14-1030

[44] Fei T , Li W , Peng J , Xiao T , Chen C‐H , Wu A , Huang J , Zang C , Liu XS & Brown M (2019) Deciphering essential cistromes using genome‐wide CRISPR screens. Proc Natl Acad Sci USA 116, 25186–25195. 31727847 10.1073/pnas.1908155116 PMC6911175

[45] Nguyen HM , Vessella RL , Morrissey C , Brown LG , Coleman IM , Higano CS , Mostaghel EA , Zhang X , True LD , Lam H‐M *et al*. (2017) LuCaP prostate cancer patient‐derived xenografts reflect the molecular heterogeneity of advanced disease an–d serve as models for evaluating cancer therapeutics. Prostate 77, 654–671. 28156002 10.1002/pros.23313 PMC5354949

[46] Aryee MJ , Liu W , Engelmann JC , Nuhn P , Gurel M , Haffner MC , Esopi D , Irizarry RA , Getzenberg RH , Nelson WG *et al*. (2013) DNA methylation alterations exhibit intraindividual stability and interindividual heterogeneity in prostate cancer metastases. Sci Transl Med 5, 169ra10. 10.1126/scitranslmed.3005211 PMC3577373 23345608

[47] Brannon AR & Sawyers CL (2013) “N of 1” case reports in the era of whole‐genome sequencing. J Clin Invest 123, 4568–4570. 24135144 10.1172/JCI70935 PMC3809802

[48] Beltran H , Hruszkewycz A , Scher HI , Hildesheim J , Isaacs J , Yu EY , Kelly K , Lin D , Dicker A , Arnold J *et al*. (2019) The role of lineage plasticity in prostate cancer therapy resistance. Clin Cancer Res 25, 6916–6924. 31363002 10.1158/1078-0432.CCR-19-1423 PMC6891154

[49] Bluemn EG , Coleman IM , Lucas JM , Coleman RT , Hernandez‐Lopez S , Tharakan R , Bianchi‐Frias D , Dumpit RF , Kaipainen A , Corella AN *et al*. (2017) Androgen receptor pathway‐independent prostate cancer is sustained through FGF signaling. Cancer Cell 32, 474–489.e6. 29017058 10.1016/j.ccell.2017.09.003 PMC5750052

[50] Rickman DS , Beltran H , Demichelis F & Rubin MA (2017) Biology and evolution of poorly differentiated neuroendocrine tumors. Nat Med 23, 1–10. 10.1038/nm.4341 28586335

[51] Aggarwal R , Huang J , Alumkal JJ , Zhang L , Feng FY , Thomas GV , Weinstein AS , Friedl V , Zhang C , Witte ON *et al*. (2018) Clinical and genomic characterization of treatment‐emergent small‐cell neuroendocrine prostate cancer: a multi‐institutional prospective study. J Clin Oncol 36, 2492–2503. 29985747 10.1200/JCO.2017.77.6880 PMC6366813

[52] Vlachostergios PJ , Puca L & Beltran H (2017) Emerging variants of castration‐resistant prostate cancer. Curr Oncol Rep 19, 32. 28361223 10.1007/s11912-017-0593-6 PMC5479409

[53] Labrecque MP , Coleman IM , Brown LG , True LD , Kollath L , Lakely B , Nguyen HM , Yang YC , da Costa RMG , Kaipainen A *et al*. (2019) Molecular profiling stratifies diverse phenotypes of treatment‐refractory metastatic castration‐resistant prostate cancer. J Clin Invest 130, 4492–4505. 10.1172/JCI128212 PMC6763249 31361600

[54] Beltran H , Prandi D , Mosquera JM , Benelli M , Puca L , Cyrta J , Marotz C , Giannopoulou E , Chakravarthi BVSK , Varambally S *et al*. (2016) Divergent clonal evolution of castration‐resistant neuroendocrine prostate cancer. Nat Med 22, 298–305. 26855148 10.1038/nm.4045 PMC4777652

