# Supplementary figures and images for: Epigenetic and transcriptional analysis reveals a core transcriptional program conserved in clonal prostate cancer metastases
Source: Mol Oncol. 2021 Mar 11;15(7):1942–55. doi: 10.1002/1878-0261.12923 (PMC8253095; doi:10.1002/1878-0261.12923)

# Supplemental Figure 1. ChIP-seq quality measures of anatomically distinct metastases.

A

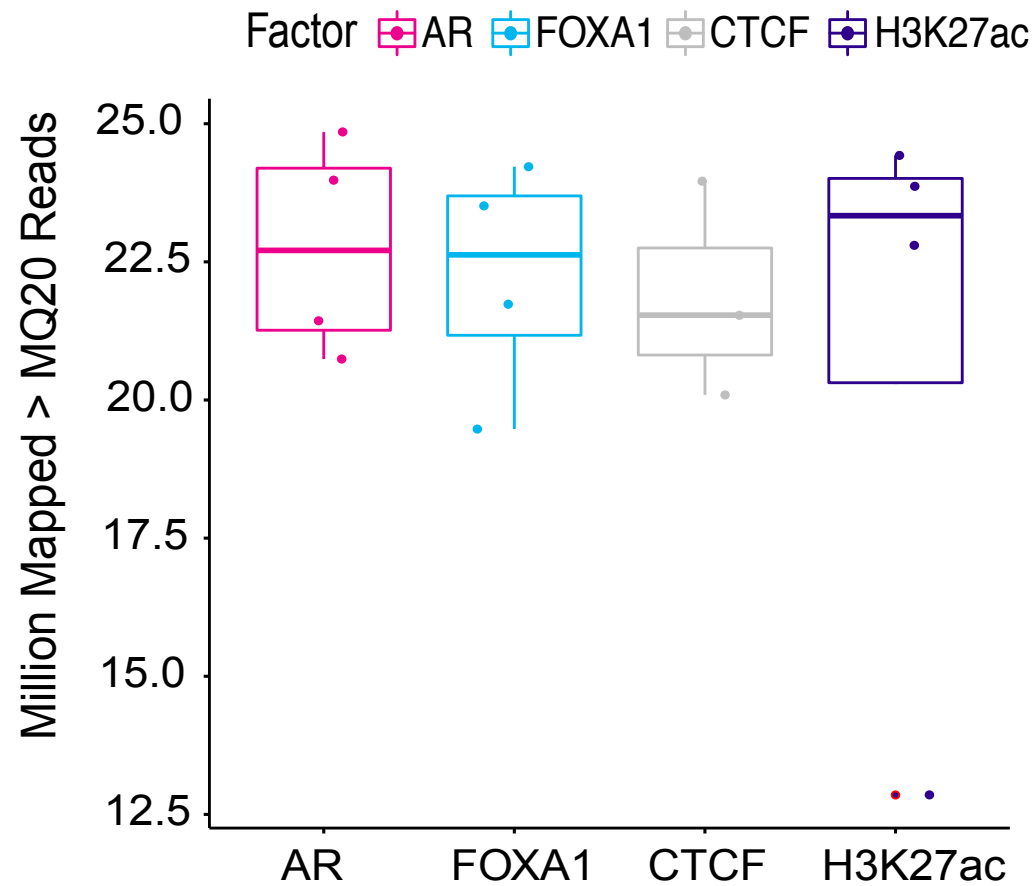

B

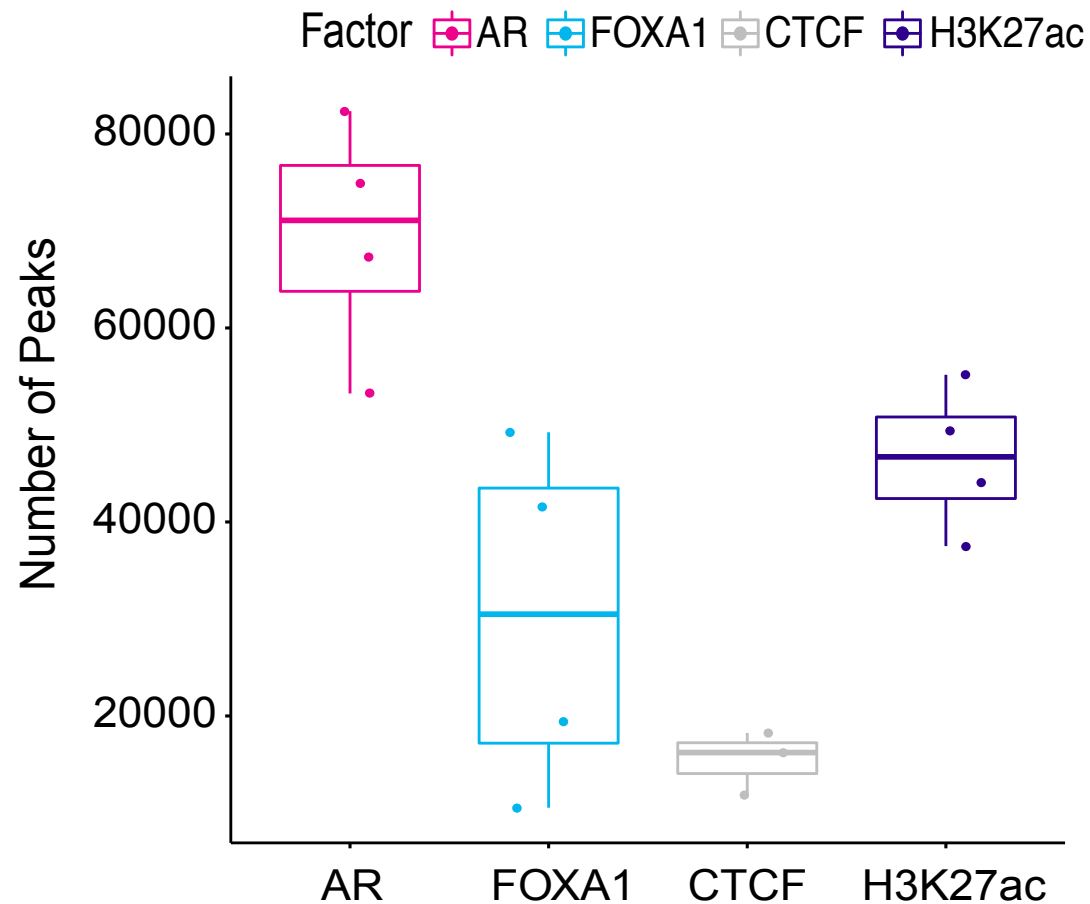

Supplement: Supplementary file 1 — Fig. S1. ChIP‐seq quality measures of anatomically distinct metastases. (A) Boxplots of millions of mapped reads > MQ20 for AR (pink), FOXA1 (turquoise), CTCF (gray) and H3K27ac (dark blue). AR, FOXA1 and H3K27ac have n = 4. CTCF has n = 3. The one outlier H3K27ac sample (outlined in red) indicates the data are less than the 25th percentile (Q1) ‐ 1.5*(interquartile range). (B) Boxplots of number of peaks of AR (pink), FOXA1 (turquoise), CTCF (gray) and H3K27ac (dark blue). Number of samples is the same as left plot. [file MOL2-15-1942-s001.pdf]
