# Supplementary material for: Epigenetic and transcriptional analysis reveals a core transcriptional program conserved in clonal prostate cancer metastases
Source: Mol Oncol. 2021 Mar 11;15(7):1942–55. doi: 10.1002/1878-0261.12923 (PMC8253095; doi:10.1002/1878-0261.12923)

Supplemental Figure 2. Shared and unique chromatin binding sites of AR, FOXA1 and CTCF.

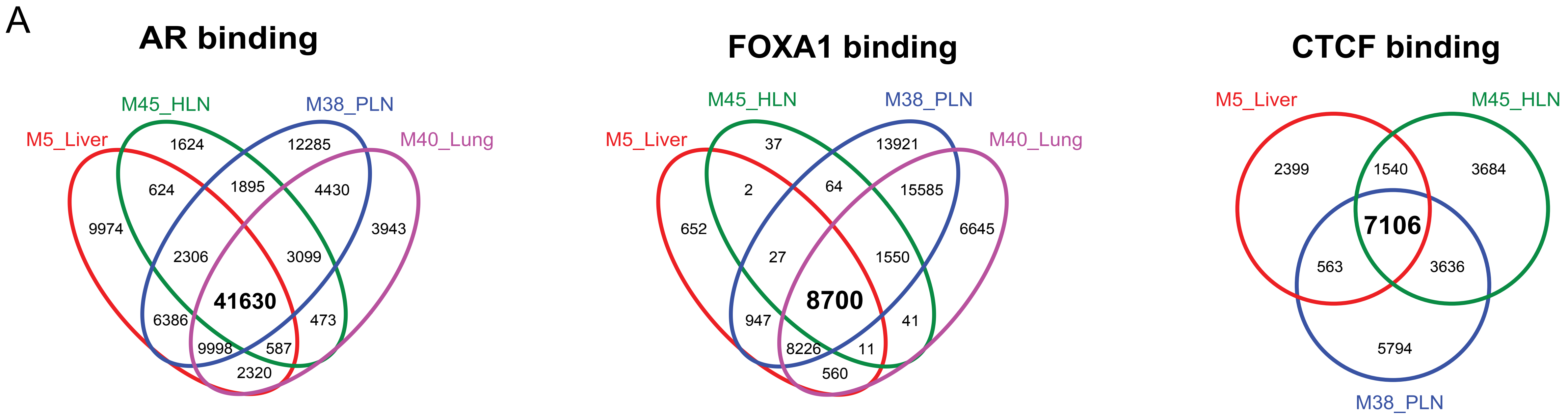

Supplement: Supplementary file 2 — Fig. S2. Shared and unique chromatin binding sites of AR and FOXA1. (A) Venn diagrams depicting the number of shared and unique AR (left), FOXA1 (center) and CTCF (right) binding sites in 3 ‐ 4 anatomically distinct metastases. [file MOL2-15-1942-s006.pdf]
