# Supplementary material for: Epigenetic and transcriptional analysis reveals a core transcriptional program conserved in clonal prostate cancer metastases
Source: Mol Oncol. 2021 Mar 11;15(7):1942–55. doi: 10.1002/1878-0261.12923 (PMC8253095; doi:10.1002/1878-0261.12923)

Supplemental Figure 3. Overlap of LNCaP essential genes and genes associated with H3K27ac-positive sample-shared AR sites

A

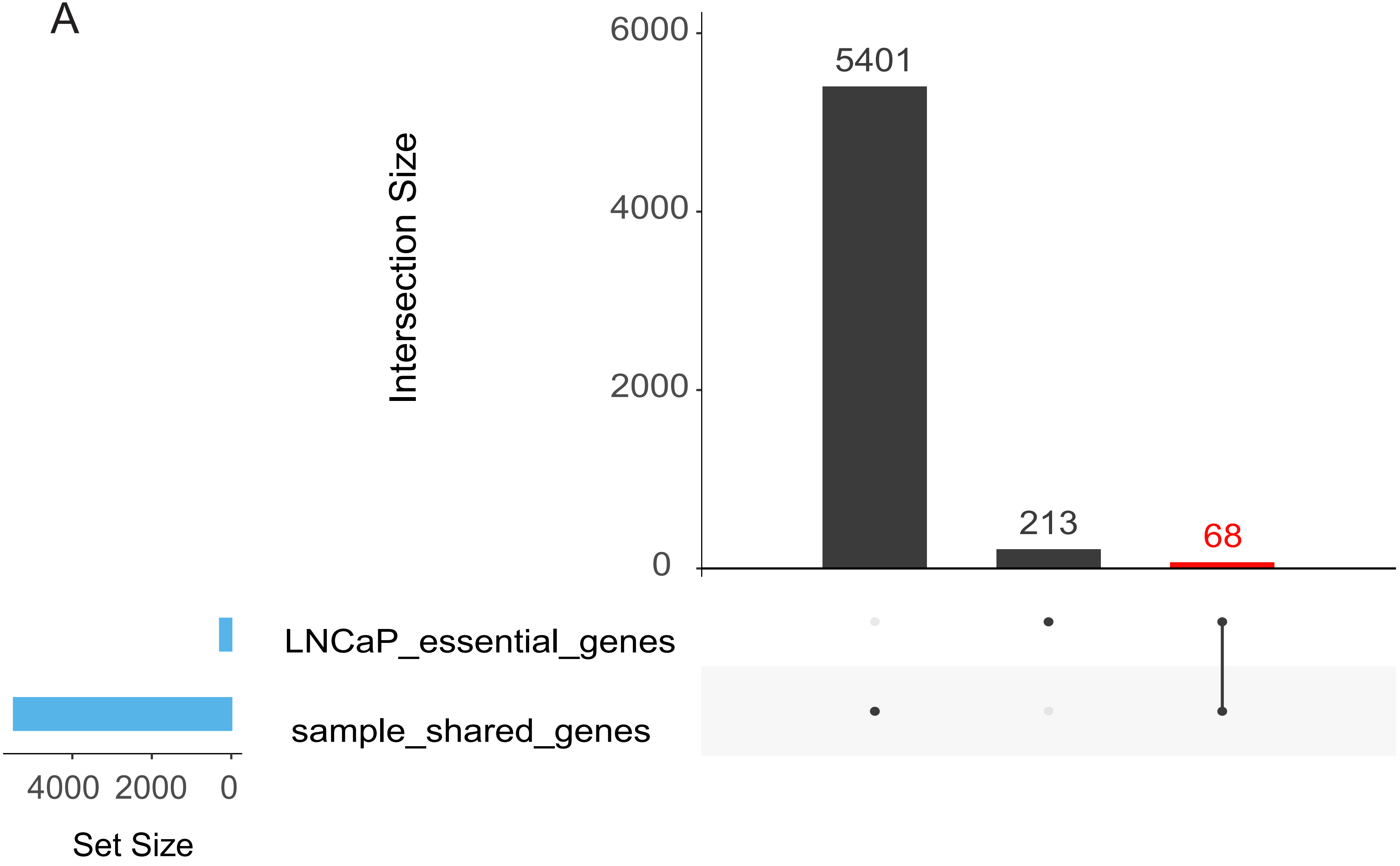

Supplement: Supplementary file 3 — Fig. S3. Overlap of LNCaP essential genes and genes associated with active sample‐shared AR sites. (A) UpSetR diagram depicting number of shared (red) and unique (gray) genes in LNCaP essential genes [47] and genes associated with active (H3K27ac‐positive) sample‐shared AR sites. [file MOL2-15-1942-s002.pdf]
