# Supplementary material for: Epigenetic and transcriptional analysis reveals a core transcriptional program conserved in clonal prostate cancer metastases
Source: Mol Oncol. 2021 Mar 11;15(7):1942–55. doi: 10.1002/1878-0261.12923 (PMC8253095; doi:10.1002/1878-0261.12923)

Supplemental Figure 4. Percentage and overlap of genes in significantly enriched Hallmark gene sets and gene set expression.

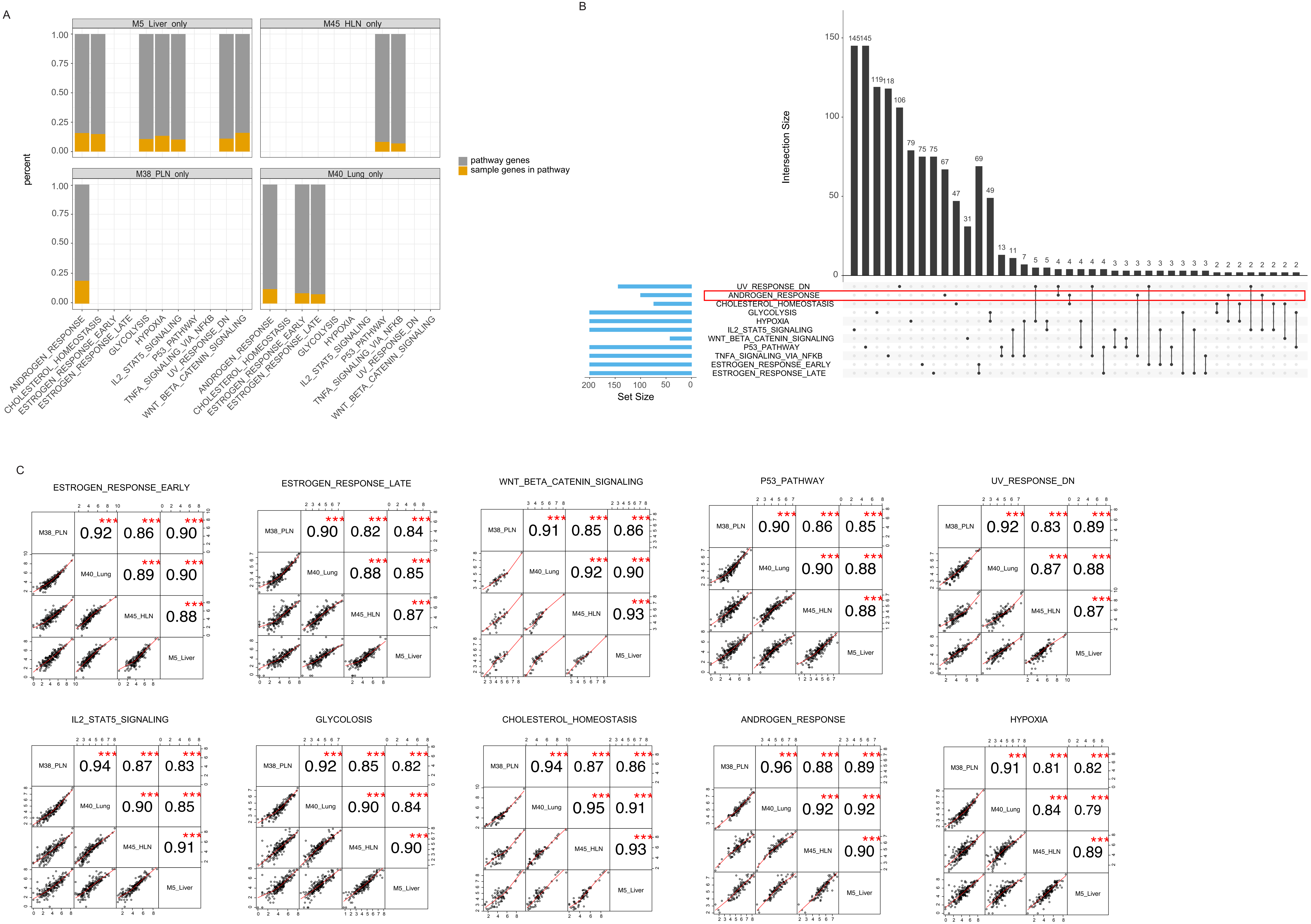

Supplement: Supplementary file 4 — Fig. S4. Percentage and overlap of genes in significantly enriched Hallmarks gene sets and gene set expression correlation. (A) Stacked barplots indicating the percentage of genes which were in sample‐specific lists (orange) in significantly enriched gene sets (gray). (B) UpSetR diagram depicting the shared and unique genes in Hallmarks gene sets that were sample‐specifically significantly enriched. Red box indicates overlaps of genes in gene sets with the Androgen Response gene set. (C) Correlation matrix of normalized log‐transformed gene expression data for remaining Hallmark gene sets for all samples. Pearson correlation and significance are indicated in the top right (*** indicates P < 0.0001). [file MOL2-15-1942-s003.pdf]
